# Effects of exercise on the levels of BDNF and executive function in adolescents: A protocol for systematic review and meta-analysis

**DOI:** 10.1097/MD.0000000000016445

**Published:** 2019-07-12

**Authors:** Kesley Pablo Morais de Azevedo, Victor Hugo de Oliveira Segundo, Gidyenne Christine Bandeira Silva de Medeiros, Ádala Nayana de Sousa Mata, Daniel Ángel García, José Carlos Gomes de Carvalho Leitão, Maria Irany Knackfuss, Grasiela Piuvezam

**Affiliations:** a Department of Public Health, Federal University of Rio Grande do Norte, Natal/RN; b University of Murcia, Department of Sociosanitary Sciences, Murcia, Spain; c Center for research in sport, health and human development, University of Trás-os-Montes and Alto Douro, Vila Real, Portugal; d Post-Graduate program in health and society, University of the State of Rio Grande do Norte (UERN), Mossoró/RN, Brazil.

**Keywords:** adolescent, BDNF, executive function, exercise, systematic review

## Abstract

**Backoground:**

Evidence available in the literature suggests that physical exercise increases the release of brain-derived neurotrophic factor (BDNF) in humans and may possibly be related to improvements in executive function. However, studies of this phenomenon in adolescents are still scarce. The objective of this work is to describe the protocol for a systematic review (SR) and meta-analysis of interventional studies aiming to determine the effect of physical exercise on BDNF levels and executive function in adolescents.

**Methods:**

This protocol is guided by the Preferred Reporting Items for Systematic Reviews and Meta-Analyses Protocols (PRISMA-P) and by the Cochrane Handbook of Systematic Reviews of Interventions. The databases to be searched are PubMed, EMBASE, Scopus, ScienceDirect, Web of Science, SPORTDiscus, the Cochrane Central Register of Controlled Trials (CENTRAL), and CINAHL. Interventional studies conducted on adolescents with different exercise protocols and evaluations of BDNF levels and executive function in pre- and post-intervention periods will be included in the systematic review. The characteristics of the studies, participants, and main results will be described, then the evaluation of the risk of biases and the level of evidence obtained by the protocol will be assessed. The selection of studies, data extraction, and evaluation of the methodological quality will be performed by 2 experienced reviewers independently.

**Conclusion:**

The systematic review will present the effects of the practice of physical exercises on the BDNF and executive function levels. The results will strengthen the interventions with the focus on the brain health of adolescents through general orientations and the evidences described shall direct future research.

**PROSPERO registration number:**

CRD42018110683

## Abstract 2

Supplemental Digital Content is available in the text

## 1 Introduction

Adolescence is marked by biological, behavioral, and social changes that gradually progress with brain development.^[1]^ Researching methods that potentially affect brain health with the prospect of improving cognitive performance, academic performance, and professional trajectory is indispensable.^[2,3]^

At the adolescent stage of human life, while the prefrontal cortex (CPF) is still maturing, the process of neural reorganization and cognitive development is perceptible.^[4]^ There is a sharp increase in brain plasticity, and according to current evidence, physical activities may stimulate some markers—including brain-derived neurotrophic factor (BDNF)—that are related to these changes.^[5]^

BDNF is a protein that strengthens neural connectivity and plays a relevant role in angiogenesis.^[6]^ Investigations have suggested a positive dose-response relationship between acute exercise and BDNF concentrations.^[7]^ In addition, studies indicate that physical exercise often increases BDNF in adults;^[8]^ however, there is a lack of evidence regarding the effects of physical exercise on BDNF in adolescent populations.

The release of BNDF in humans is stimulated by physical activity and may be related to improvements in executive function.^[9,10]^ Executive function is responsible for higher cognitive processes involved in managing other basic cognitive functions; additionally, it guides decision-making processes for motor tasks and healthy behaviors.^[11–13]^

In order to understand the effects of physical exercise on cognitive development during adolescence, intervention studies have been performed with different physical exercise programs that evaluated the effects of physical activity on BDNF levels and executive function.^[2,14–17]^ From these studies, the observation was made that alterations in the BDNF levels that are induced by physical exercise may be related to the intensity of the exercises.^[11]^

Thus, several studies have examined BDNF and cognitive function while considering various types of exercises in humans; however, results in adolescents are still scarce.^[7–10]^ Considering the variability of physical exercise programs adopted in interventions and their effects on the levels of BDNF and executive function, it is an opportune time to develop a systematic review protocol and meta-analysis on the effects of different physical exercise programs in the levels of BDNF and executive function in the adolescent population.

### 1.1 Objective

Describe the protocol of a systematic review and meta-analysis of clinical trials that determine the effects of physical exercise on the levels of BDNF and executive function in adolescents.

## 2 Methods

### 2.1 Protocol and registration

This protocol was prepared in accordance with the guidelines described by the Preferred Reporting Items for Systematic Reviews and Meta-Analyses Protocols (PRISMA-P)^[18]^ and the Cochrane Handbook of Systematic Reviews of Interventions.^[19]^

The protocol was registered with the International Prospective Register of Systematic Reviews (PROSPERO) on 30 November 2018 (CRD42018110683).

### 2.2 Eligibility criteria

#### 2.2.1 Inclusion criteria

In this review, peer-reviewed journal articles that meet the eligibility criteria based on the study population, interventions, control, and outcome (PICOS) will be included.^[20,21]^

The eligibility criteria for inclusion are as follows: clinical trial studies; studies performed with adolescents of both sexes, ages 10 to 19 years; studies that contain aerobic exercise programs, resistance training, or concurrent training (aerobic and resistance training) with consideration to variations in frequency, duration, and intensity; and studies that assess BDNF levels and executive function.

#### 2.2.2 Exclusion criteria

Studies that used adolescents with physical or intellectual disabilities or with psychological or neuroendocrine problems will be excluded.

### 2.3 Information sources and literature search

Initially for the identification of the appropriate studies, search strategies will be developed from keywords indexed in the Medical Subject Headings (MeSH), a combination of descriptors related to the adolescent study population, the exercise program intervention, and the BDNF and executive function outcomes under consideration. These will be accompanied by the Boolean operators OR and AND (Table 1). Next, search strategies will be applied in the following electronic databases: EMBASE, Scopus, ScienceDirect, Web of Science, SPORTDiscus, the Cochrane Central Register of Controlled Trials (CENTRAL), CINAHL, and PubMed (Appendix I). The searches in these databases will be performed on a computer with the IP of the Federal University of Rio Grande do Norte (UFRN), which gives open access to all articles in each database through the “Portal de Periodicos Capes” in Brazil. The EMBASE search will be performed in the University of São Paulo (USP).

**Table 1:**
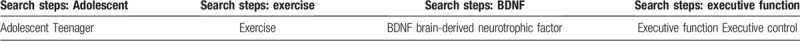
Search strategies.

In regard to gray literature, searches will be carried out in repositories for dissertations and theses, searches for abstracts and contact with authors who research this subject will be conducted when the need for these searches is identified. Bibliographic searches will be carried out by 2 researchers. The initial searches will test preliminary equations with the perspective of applying search strategies with high sensitivity to the subject.

**Figure 1:**
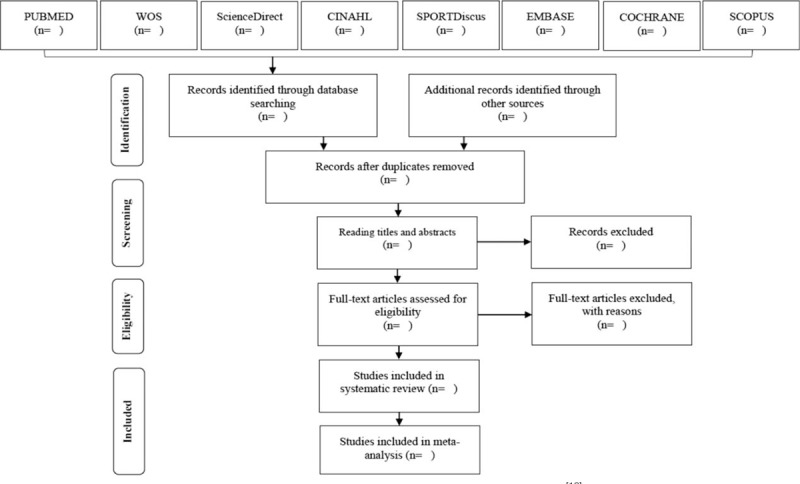
Article selection flowchart. Adapted from PRISMA-P^[18]^.

At the end of the database searches, the articles will be compiled into the EndNote bibliographical reference manager, and the duplicate articles will be removed. Subsequently, the reading of the titles and abstracts will be carried out by 2 researchers who shall select the articles according to the eligibility criteria. Soon after, the full text of the articles and their references will be analyzed, and the studies that are appropriate under the inclusion criteria of SR will be selected (Fig. 1). Any discrepancies that occur during the screening phases, such as assessing eligibility for full-text analysis and screening titles and abstracts, will be resolved with the help of a third researcher.

### 2.4 Data extraction

Two reviewers will be responsible for extracting and managing the data, which will be inserted into an EXCEL spreadsheet; doubts will be clarified with the help of the third researcher.

Information on the characteristics, methodological aspects, and main results of the selected studies will be collected as described in Table 2.

**Table 2:**
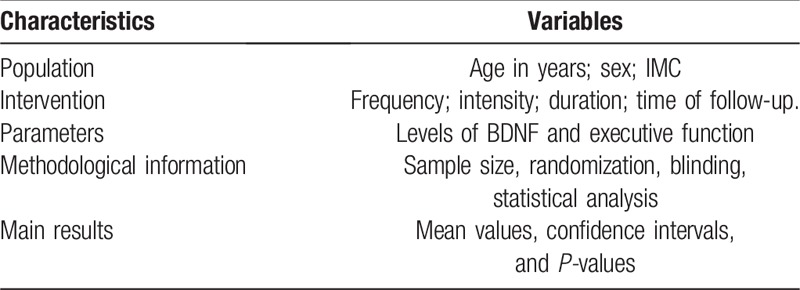
Characteristics and methodological aspects of the selected studies.

### 2.5 Risk of bias assessment and Grading of Recommendations Assessment, Development, and Evaluation (GRADE) assessment

Two reviewers will assess the risk of bias in the selected studies and any differences will be resolved by consulting a third reviewer. For the evaluation, the risk bias tool developed by Cochrane will be used.^[22]^ The reviewers will have been previously trained and calibrated to ensure uniformity in the evaluation of the criteria, and a kappa index will be applied for agreement analysis. The evaluation of the evidence and the strength of the recommendations of the studies will assessed with the Grading of Recommendations Assessment, Development, and Evaluation tool (GRADE).^[23]^

### 2.6 Data analysis and synthesis

The systematic review will describe relevant information from the included studies, such as the sample characteristics, types of interventions, instruments of measurement, primary outcomes (BDNF and executive function), and secondary outcomes (nutritional status, body composition and physical fitness), statistical analysis, and the main results.

The results corresponding to the effects of the interventions will be stated, and these will take into account the difference in averages and *P* values, for the measurements evaluated before, during, and after the interventions. The comparative analyses performed and described between intervention groups and between the intervention groups respective of their control groups will be presented.

The heterogeneity between trial results will be evaluated using a standard *χ*^2^ test with a significance level of 0.05. To assess heterogeneity, we plan to compute the *I*^2^ statistic, which is a quantitative measure of inconsistency across studies. A value of 0% indicates no observed heterogeneity, whereas *I*^2^ values of 50% indicate a substantial level of heterogeneity. For low heterogeneity, a meta-analysis of fixed effect will be used to estimate the treatment effect; for high heterogeneity, we will use the random effects model.

## 3 Discussion

The systematic review and meta-analysis proposed will present the results of studies that evaluated the effects of physical exercise interventions on BDNF levels and executive function in the adolescent population. Recently, clinical trials have been published that have tested the acute and chronic effects of aerobic training programs, strength training, and concurrent training on BDNF levels and executive function in adolescents.^[2,14,15,17]^ However, there was heterogeneity among the programs regarding the time of intervention, frequency, duration of sessions, and intensity. Analyzing these variables could support better decision making for future interventions and health programs aimed at adolescents.

The results of a systematic review conducted with healthy adults identified that peripheral concentrations of BDNF levels increase after interventions, regardless of whether the aerobic exercises were acute or chronic; these findings corroborate the hypothesis that physical exercise increases BDNF levels and, therefore, executive function.^[10]^ In an attempt to clarify the effects of exercise on the levels of BDNF and its relationship with gains in cognitive development, the authors indicate the need to perform new analyses in other age groups, with a standardization of the types of studies, types of interventions, and types of instruments applied.

A meta-analysis that addressed 55 studies with 1180 participants (75.4% male with a mean age of 27.9 ± 10.8 years) identified positive results in the increase of BDNF levels after a single exercise session. These results suggest that the effects of the interventions may be related to the duration and intensity of the exercises, and they may also be different between sexes.^[8]^

Regarding younger populations, a meta-analysis of 24 clinical trials with a total of 948 participants showed improvements in the executive function of children, adolescents, and young adults undergoing aerobic physical exercise programs. The results are favorable for interventions that tested acute and chronic effects; however, only 2 studies were performed with adolescents, and they only focused on inhibitory control.^[24,25]^ To address the relevance of studies aimed at healthy brain development, researchers warn of the damage caused by a sedentary lifestyle and lifestyle factors that cause obesity.^[26]^

Similar results were observed in a systematic review where adolescents undergoing aerobic exercise programs obtained improvements in cognitive function; in that review, 10 studies that evaluated 588 healthy adolescents (62.9% males) aged 13 to 16 years were included. Improvements in cognitive function were observed in programs for acute and chronic physical exercises; ^[11]^ however, the evidence is limited because of the heterogeneity of the studies; variability of the instruments applied; and the differences in frequency, intensity, and duration of the programs.

As indicated previously, it has been observed that adolescents undergoing physical exercise programs have improved cognitive function domains. However, the results are scarce regarding the impact of these interventions on levels of BDNF and executive function. The evidence available in the literature deals with the effects of exercise on BDNF levels in other age groups. From this perspective, this protocol intends to overcome these limitations by analyzing subgroups while taking into account the heterogeneity of interventions, variability of the instruments of measurement applied, and the characteristics of the participants.

The protocol for this systematic review and meta-analysis is presented in a clear and systematic way for the extraction of information and presentation of relevant results on the effects of physical exercise on the levels of BDNF and executive function in adolescents.

## Author contributions

**Conceptualization:** Grasiela Piuvezam.

**Data curation:** Kesley Pablo Morais de Azevedo, Victor Hugo de Oliveira Segundo, Gidyenne Christine Bandeira Silva de Medeiros, Daniel Ángel García.

**Formal analysis:** Kesley Pablo Morais de Azevedo, Gidyenne Christine Bandeira Silva de Medeiros, Ádala Nayana de Sousa Mata, Daniel Ángel García, Grasiela Piuvezam.

**Funding acquisition:** Kesley Pablo Morais de Azevedo.

**Investigation:** Kesley Pablo Morais de Azevedo.

**Methodology:** Kesley Pablo Morais de Azevedo, Gidyenne Christine Bandeira Silva de Medeiros, Daniel Ángel García, José Carlos Gomes de Carvalho Leitão, Maria Irany Knackfuss, Grasiela Piuvezam.

**Project administration:** Kesley Pablo Morais de Azevedo, Grasiela Piuvezam.

**Resources:** Kesley Pablo Morais de Azevedo, Victor Hugo de Oliveira Segundo, Daniel Ángel García.

**Software:** Kesley Pablo Morais de Azevedo.

**Supervision:** Grasiela Piuvezam.

**Writing – original draft:** Kesley Pablo Morais de Azevedo.

**Writing – review & editing:** Ádala Nayana de Sousa Mata, Daniel Ángel García, José Carlos Gomes de Carvalho Leitão, Maria Irany Knackfuss, Grasiela Piuvezam.

## Supplementary Material

Supplemental Digital Content
