# Supplementary material for: Effects of exercise on the levels of BDNF and executive function in adolescents: A protocol for systematic review and meta-analysis
Source: Medicine (Baltimore). 2019 Jul 12;98(28):e16445. doi: 10.1097/MD.0000000000016445 (PMC6641795; doi:10.1097/MD.0000000000016445)
Supplement: Supplemental Digital Content [file medi-98-e16445-s001.docx]

**Appendix I**. Search strategy for PUBMED database.

| **PUMED** | | | | | | |
| --- | --- | --- | --- | --- | --- | --- |
| ("exercise") | AND | ("BDNF" OR "Brain Derived Neurotrophic Factor”) | AND | ("Executive function" OR "Executive Control") | AND | ("adolescent" OR "teenager") |
